# Deciphering the possible role of *ctxB7* allele on higher production of cholera toxin by Haitian variant *Vibrio cholerae* O1

**DOI:** 10.1371/journal.pntd.0008128

**Published:** 2020-04-01

**Authors:** Arindam Naha, Rahul Shubhra Mandal, Prosenjit Samanta, Rudra Narayan Saha, Sreeja Shaw, Amit Ghosh, Nabendu Sekhar Chatterjee, Pujarini Dutta, Keinosuke Okamoto, Shanta Dutta, Asish Kumar Mukhopadhyay

**Affiliations:** 1 Division of Bacteriology, ICMR-National Institute of Cholera and Enteric Diseases, Kolkata, India; 2 Biomedical Informatics Center, ICMR-National Institute of Cholera and Enteric Diseases, Kolkata, India; 3 Division of Biochemistry, ICMR-National Institute of Cholera and Enteric Diseases, Kolkata, India; 4 Division of Clinical Medicine, ICMR-National Institute of Cholera and Enteric Diseases, Kolkata, India; 5 Collaborative Research Center of Okayama University for Infectious Diseases at NICED, Kolkata, India; Johns Hopkins Bloomberg School of Public Health, UNITED STATES

## Abstract

Cholera continues to be an important public health concern in developing countries where proper hygiene and sanitation are compromised. This severe diarrheal disease is caused by the Gram-negative pathogen *Vibrio cholerae* belonging to serogroups O1 and O139. Cholera toxin (CT) is the prime virulence factor and is directly responsible for the disease manifestation. The *ctxB* gene encodes cholera toxin B subunit (CTB) whereas the A subunit (CTA) is the product of *ctxA* gene. Enzymatic action of CT depends on binding of B pentamers to the lipid-based receptor ganglioside G_M1_. In recent years, emergence of *V*. *cholerae* Haitian variant strains with *ctxB7* allele and their rapid spread throughout the globe has been linked to various cholera outbreaks in Africa and Asia. These strains produce classical type (WT) CTB except for an additional mutation in the signal sequence region where an asparagine (N) residue replaces a histidine (H) at the 20^th^ amino acid position (H20N) of CTB precursor (pre-CTB). Here we report that Haitian variant *V*. *cholerae* O1 strains isolated in Kolkata produced higher amount of CT compared to contemporary O1 El Tor variant strains under *in vitro* virulence inducing conditions. We observed that the *ctxB7* allele, itself plays a pivotal role in higher CT production. Based on our *in silico* analysis, we hypothesized that higher accumulation of toxin subunits from *ctxB7* allele might be attributed to the structural alteration at the CTB signal peptide region of pre-H20N CTB. Overall, this study provides plausible explanation regarding the hypertoxigenic phenotype of the Haitian variant strains which have spread globally, possibly through positive selection for increased pathogenic traits.

## Author summary

Cholera remains to be a major public health burden in the developing countries. Toxigenic strains of the Gram-negative organism *Vibrio cholerae* belonging to serogroups O1 and O139 are the etiological agents for this dreadful disease. These strains secrete CT into the extracellular milieu which upon ingestion of contaminated food or water, results in severe loss of water and electrolytes. In the recent past, *V*. *cholerae* O1 strains with the signature *ctxB7* allele have propagated through many of the cholera endemic regions in Africa and Asia. These strains were also identified as the cause for the devastating cholera epidemic in Haiti that killed around 8000 victims. It was demonstrated that the Haitian isolate had been associated with hypertoxigenic phenotype and increased virulence. However, whether the *ctxB7* allele may have any role to play in increased CT production phenotype of the Haitian variants remains obscure. We observed that the *ctxB7* allele plays a pivotal role in heightened CT production, although any link between increased CT production and hypervirulence phenotypes remains undefined.

## Introduction

Since 1817, cholera has spread into many countries and over the years has caused several pandemics [1]. It is now well established that the classical biotype was responsible for the fifth and sixth pandemics whereas the ongoing seventh pandemic is due to El Tor biotype [2]. These strains have spread globally in three separate waves [3]. While acquiring numerous changes in their genetic background, wave 3 altered El Tor (AET) strains have propagated through many of the cholera endemic regions in Asia and Africa [4]. After the 2010 earthquake in Haiti, a disastrous cholera outbreak emerged in late October which affected over 600,000 people, resulting in deaths of around 8000 Haitians [5]. Previously, emergence and dissemination of *V*. *cholerae* O1 strains with *ctxB7* allele was reported from cholera outbreaks in India. [6–7]. Furthermore, clonal variants of *V*. *cholerae* O1 El Tor related to the Haitian cholera epidemic (henceforth designated as “Haitian variant”) have been linked to Cameroon, Nepal and Nigeria [8–10]. Apart from the presence of additional mutations in the toxin co-regulated pilus subunit A (*tcpA*^*CIRS*^) and a null mutation in the *rtxA* gene encoding a truncated multifunctional auto processing RTX (MARTX) family toxin [11], these Haitian variant strains also harbor *ctxB7* allele which results in H20N substitution in the CTB signal sequence [12]. However, the CT epitype remains identical to CT1 as the mutated signal peptide (H20NCTB_SS_) is processed by the membrane bound signal peptidase, releasing the mature portion of the precursor CTB protein (pre-CTB) in to the periplasmic space during pre-CTB translocation across the bacterial cytoplasmic membrane. The mature CTB monomers which are identical to CTB classical type then assemble into pentamers and associate with A subunits to produce holotoxin [13].

Previous studies support that *V*. *cholerae* O1 strains with the *ctxB7* allele produce excess CT compared to prototypical El Tor isolates [4, 14]. This excess toxin production has been attributed in part to change in virulence gene regulation due to single nucleotide substitutions in *hns* [15] encoding the histone-like nucleoid structuring protein (H-NS) and *vieA* [16], encoding a cyclic diguanylic acid (c-di-GMP) phosphodiesterase (PDE). Furthermore, Haitian outbreak strain had also been found to exhibit characteristics of hypervirulence with increased pathogenic potential [4]. Recently, one of the reports from our group has shown that Haitian variant strains of *Vibrio cholerae* O1 manifested markedly higher fluid accumulation and increased mucosal damage in animal models [17]. However, it was not previously known whether the *ctxB7* allele may have contributed to hypertoxigenic phenotype of these atypical variants isolates. Against this backdrop, it was thought worthwhile to examine the CT production by these strains and to examine the role, if any, of the *ctxB*7 allele in this process.

In this article we report that the Haitian *ctxB* plays a definite crucial role in heightened CT production. We also propose a hypothesis which suggests a plausible role of the altered CTB signal sequence (H20NCTB_SS_) in efficient pre-CTB translocation and processing across the inner membrane, although it remains to be proven experimentally. Our results are consistent with previous reports that Haitian variant isolates produces excess CT. Finally, this study indicates that *ctxB7* allele may have a role to play in the hypertoxigenic phenotype of Haitian variant strains. However, whether the increased CT production from the *ctxB7* allele may have a contribution to hypervirulence, it remains yet to be determined.

## Methods

### Plasmids, bacterial strains and culture conditions

Plasmids, strains, and oligonucleotides used in this study are listed in Table 1. All of the *V*. *cholerae* strains used in this study were obtained from the strain repository of the ICMR-National Institute of Cholera and Enteric Diseases (NICED), Kolkata, India. JBK70, an isogenic *ctxAB* deleted strain of N16961 [18] was obtained from Dr. James Kaper, University of Maryland, School of Medicine. *E*. *coli* K12 strain DH5α were used for all of the cloning experiments whereas LMG194 [19], a generous gift from Prof. Scott Butler, University of Rochester Medical center) was used as the host strain for arabinose inducible protein expression. All strains were maintained at -80°C as 25% glycerol stock. For toxin assays, arabinose inducible expression plasmids pBAD24 and pBAD33 [19] obtained from Dr. Rupak K. Bhadra, CSIR Indian Institute of Chemical Biology and Dr. Jeffrey H. Withey, Wayne State University School of Medicine, respectively, were used. Unless otherwise stated, bacterial strains were routinely grown on Luria agar (LA) or broth (LB) with ampicillin (100 μg/ml) or chloramphenicol (30 μg/ml) wherever needed.

**Table 1:** Bacterial plasmids, strains and primer sequences used in the study.

Plasmids or strains	Genotype and/or phenotype	Reference /source
**Plasmids**		
pBAD24	Bacterial expression vector pBR322 *ori araC bla*; Amp^r^	[19]; Dr. Rupak K. Bhadra, CSIR IICB
pBAD33	Bacterial expression vector pACYC184 *ori araC bla*; Cm^r^	[19];Dr. Jeffrey H. Withey, Wayne State University
pAN1	*ctxB* gene from *V*. *cholerae* O395 cloned with in the EcoRI- HindIII sites of pBAD24	This study
pAN2	*ctxB* gene from *V*. *cholerae* 2010EL-1786 cloned with in the EcoRI- HindIII sites of pBAD24	This study
pAN3	*ctxA* gene from *V*. *cholerae* O395 cloned with in the PstI- HindIII sites of pBAD33	This study
**Strains**		
***Vibrio cholerae***	**O1 Variants**	
K3314	*V*. *cholerae* O1, El Tor Variant, 2005 isolate	Laboratory collection
L4867	*V*. *cholerae* O1, El Tor Variant, 2006 isolate	Laboratory collection
IDH00161	*V*. *cholerae* O1, El Tor Variant, 2007 isolate	Laboratory collection
IDH00790	*V*. *cholerae* O1, El Tor Variant, 2008 isolate	Laboratory collection
IDH03371	*V*. *cholerae* O1, El Tor Variant, 2010 isolate	Laboratory collection
L19494	*V*. *cholerae* O1, Haitian Variant, 2006 isolate	Laboratory collection
IDH00990	*V*. *cholerae* O1, Haitian Variant, 2008 isolate	Laboratory collection
IDH03311	*V*. *cholerae* O1, Haitian Variant, 2010 isolate	Laboratory collection
IDH03595	*V*. *cholerae* O1, Haitian Variant, 2011 isolate	Laboratory collection
IDH03454	*V*. *cholerae* O1, Haitian Variant, 2011 isolate	Laboratory collection
2010EL-1786	*V*. *cholerae* O1, Haitian outbreak strain	Laboratory collection
	**Prototype El Tor/Classical**	
V24	*V*. *cholerae* O1, El Tor, 1989 isolate	[20]; Laboratory collection
V7	*V*. *cholerae* O1, El Tor, 1989 isolate	[20]; Laboratory collection
V32	*V*. *cholerae* O1, El Tor, 1989 isolate	[20]; Laboratory collection
V54	*V*. *cholerae* O1, El Tor, 1989 isolate	[20]; Laboratory collection
V100	*V*. *cholerae* O1, El Tor, 1990 isolate	[20]; Laboratory collection
GP15	*V*. *cholerae* O1, Classical isolate	[20]; Laboratory collection
GP145	*V*. *cholerae* O1, Classical isolate	[20]; Laboratory collection
GP147	*V*. *cholerae* O1, Classical isolate	[20]; Laboratory collection
L362	*V*. *cholerae* O1, Classical isolate	[20]; Laboratory collection
569B	*V*. *cholerae* O1, Classical strain	Laboratory collection
	**Recombinant strains**	
JBK70	N16961 *ctxA*^*-*^*ctxB*^-^	[18]; Dr. James Kaper, University of Maryland
AN1	JBK70 containing pBAD24, Amp^R^	This study
AN2	JBK70 containing pAN1, Amp^R^, encodes WTCTB_SS_	This study
AN3	JBK70 containing pAN3, Amp^R^, encodes H20NCTB_SS_	This study
***Eschericia coli***		
DH5α	F^–^ φ80*lac*ZΔM15Δ(*lac*ZYA-*arg*F)U169 *rec*A1 *end*A1 *hsd*R17(r_K_^–^, m_K_^+^) *pho*A *sup*E44 λ^–^ *thi*-1 *gyr*A96 *rel*A1	Laboratory collection
LMG194	F^-^ Δ*lacX74 galE thi rpsL* Δ*phoA (Pvu II)* Δ*ara714 leu*::Tn*10*	[19] Prof. Scott Butler, University of Rochester Medical Center
AN4	LMG194 containing pBAD24, Amp^R^	This study
AN5	LMG194 containing pAN1, Amp^R^, encodes WTCTB_SS_	This study
AN6	LMG194 containing pAN2, Amp^R^, encodes H20NCTB_SS_	This study
AN7	LMG194 containing pBAD24 and pBAD33, Amp^R^, Cm^R^	This study
AN8	AN5 containing pBAD33, Amp^R^, Cm^R^, encodes WTCTB_SS_	This study
AN9	AN6 containing pBAD33, Amp^R^, Cm^R^, encodes H20NCTB_SS_	This study
AN10	AN5 containing pAN3, Amp^R^, Cm^R^, encodes WTCTB_SS_ along with CTA	This study
AN11	AN6 containing pAN3, Amp^R^, Cm^R^, encodes H20NCTB_SS_ along with CTA	This study
AN12	JBK70 expressing pre-CTB K3E,Amp^R^	This study
**Oligonucleotide primes**		
**Primer name**	**(5′-3′) sequence**	**Reference /source**
ctxB EcoRI F	gaccgcgaattcagcatgattaaattaaaatttggtg	This study
ctxB HindIII R	cctgaagcttaatttgccatactaattgc	This study
pBAD F	cgtcacactttgctatgccatag	This study
pBAD R	ttctgttttatcagaccgcttc	This study
ctxA PstI F	gatacctgcagatggtaaagataatatttgtg	This study
ctxA HindIII R	cctgaagctttcataattcatccttaattc	This study
ctxA F RT	acggctcttccctccaagctct	This study
ctxA R RT	ggtatcgagttcattttggggtgc	This study
ctxB F RT	cctcagggtatccttcatcct	This study
ctxB R RT	gtgcagaataccacaacacac	This study
recAF	aagcaatgcgtaaactga	[21]
recAR	ggcgaatatccaaacgaa	
ctxB (F)	ggttgcttctcatcatcgaaccac	[20]
ctxB (R)	gatacacataatagaattaaggat	
zotF(S)	cgagctaccgctacaaggtgcta	[20]
ctxAR(S)	cgtgcctaacaaatcccgtctgag	
EPEC 16srRNA FP	tcgtcagctcgtgttgtgaa	[22]
EPEC 16srRNA RP	cgcttctctttgtatgcgcc	

### Nucleotide sequencing

PCR amplification *ctxB* locus and CTX promoter regions from *V*. *cholerae* O1 isolates was performed in 25μL reaction mixture using the primers ctxB (F)/ctxB (R) and zotF(S) and ctxAR(S), respectively (Table 1). PCR primers and conditions used have been previously described [20]. For amplification of *ctxA* or *ctxB* genes cloned in pBAD33 or pBAD24 respectively, pBAD F and pBAD R primers were used. PCR amplicons were purified using the Qiaquick PCR purification kit (QIAGEN, GmbH, Hilden, Germany) and both the strands were sequenced in an automated sequencer (ABI PRISM 3100 Genetic Analyser, Applied Biosystems, Foster city, CA, USA). After careful analysis of the data, nucleotide sequences had been deposited in GenBank.

### Development of recombinant plasmid constructs for arabinose inducible expression of B-pentamers (CTB) and Holotoxins (CT)

The *ctxB* and *ctxA* genes from *V*. *cholerae* O395 and 2010El-1786 chromosomal DNA were PCR amplified using primer pairs ctxB EcoRI F/ ctxB HindIII R and ctxA PstI F/ ctxA HindIII R (Table 1) with Phusion High-Fidelity DNA Polymerase (New England Biolabs) according to the manufacturer’s protocol. PCR products were purified by Qiaquick PCR purification kit (Qiagen, GmBH, Germany). The amplicons were cloned into pBAD24 or pBAD33 using EcoRI and HindIII (for *ctxB* cloning) or PstI and HindIII (*ctxA* cloning) sites producing plasmids pAN1, pAN2, and pAN3 (Table 1). Restriction enzymes were purchased from New England Biolabs and Thermo Fisher Scientific. Purified vector and insert DNAs were ligated in appropriate molar ratio with T4 DNA Ligase (Thermo Fisher Scientific). Following transformation, positive clones were confirmed by restriction digestion and by DNA sequencing of the recombinant plasmids. Sequence verified constructs were introduced into JBK70 and LMG194 strains by electroporation and transformation as previously described [23–24].

### Cholera toxin production assay

Twenty *V*. *cholerae* O1 strains were included in the *in-vitro* toxin measurement study (Table 1). To estimate the amount of CT expressed by *V*. *cholerae* O1 strains under *in vitro* conditions, bacteria were cultured in AKI [25] medium for 20 hours. For arabinose inducible expression of toxin subunits, *V*. *cholerae* and *E coli* strains were first grown under uninduced conditions [LB medium supplemented with 0.4% w/v D-(+) glucose] and subsequently shifted to inducing conditions [LB containing 0.002%, 0.02% or 0.2% w/v L-(+) arabinose]. After 6 hours of induction, the optical densities of the cultures were recorded at 600 nm (O.D. 600nm). GM1 ganglioside enzyme-linked immunosorbent CT assays (GM_1_ CT ELISA) were performed as described earlier [26] and CT produced per ml of culture per O.D.600 unit (ng/ml/O.D.600nm) was determined. Briefly, 96 well polystyrene plates (MaxiSorp, Nunc, Denmark) were coated with 100 ng GM_1_ (Sigma-Aldrich, St Louis, MO, USA), followed by blocking with 3% bovine serum albumin (A3803, Sigma-Aldrich, St Louis, MO, USA) in phosphate buffered saline (PBS). Culture supernatants and cell extracts of *V*. *cholerae* and *E*. *coli* cells were allowed to bind the GM_1_ residues attached to the wells. A standard curve was generated simultaneously with known concentrations of purified CTB or CT (Sigma-Aldrich, St Louis, MO, USA) wherever needed. Any unbound toxin molecule was washed off by PBS supplemented with 0.1% Tween 20 (Sigma-Aldrich, St Louis, MO, USA). Rabbit anti-cholera toxin (Sigma-Aldrich, St Louis, MO, USA) was used as the primary antibody (1:2000) while goat anti-rabbit conjugated to horseradish peroxidase (Jackson Immuno Research) used as the secondary antibody (1:8000) and 3,3′,5,5′-Tetramethylbenzidine (TMB, Sigma-Aldrich, St Louis, MO, USA) as the detecting reagent. An average of O.D. 450 values obtained from at least three individual experiments was considered to estimate the amount of CT present in the culture supernatant of each sample using the standard curve.

### Western blotting

AKI medium was used for culturing *V*. *cholerae* strains. The immunoblotting using monoclonal antibody against classical CTB (which was prepared by immunizing rats with a synthesized peptide (NTQIHTLNDKC) was done as described earlier elsewhere [20]. *E coli* cells grown in LB medium supplemented with arabinose were harvested by centrifugation and pellets were then suspended in lysis buffer (20 mM Tris-Cl pH 8.0, 300 mM NaCl, 1 mM EDTA pH 8.0, 0.1% Triton X-100) with cOmplete Protease Inhibitor (Roche Diagnostics, Mannheim, Germany) and disrupted by sonication (Sonicator 3000 Ultrasonic Liquid processor, Misonix, N.Y.,USA). After centrifugation at 13,000 rpm at 4°C for 15 min to remove any unbroken cells, the lysate was used for measurement of toxin secretion and immunoblot of relevant proteins. 20% trichloroacetic acid (TCA) was added to 400 μl of bacterial cell extracts and after 30 minutes on ice, centrifuged at 13,000 rpm for 10 minutes at 4°C. Pellets were washed with ice-cold acetone twice. Pellet fractions from each samples were boiled with 2X Laemmli sample buffer (Bio- Rad Laboratories, Hercules, CA, USA) for 5 min and subjected to 15% sodium dodecyl sulfate-polyacrylamide gel electrophoresis (SDS-PAGE). Proteins were then transferred to a PVDF membranes (Millipore, Billerica, MA, USA) using Trans-Blot SD Semi-Dry Transfer Cell (Bio- Rad Laboratories, Hercules, CA, USA). Blots were incubated for 2 hours in blocking solution (Tris-buffered saline containing 5% nonfat dry and 0.1% Tween 20) to reduce nonspecific binding. After a brief washing with TBST buffer (Tris-buffered saline containing 0.1% Tween 20), they were incubated overnight in blocking solution containing 1:10,000 dilution of mouse monoclonal anti-DnaK (8E2/2, Enzo Life Sciences) or 1:500 dilution of anti-β-lactamase (ab12251, Abcam) antibody. After three washes for a total of 30 mins with TBST buffer, each blot was incubated for 1 h in blocking buffer containing 1:5,000 dilution of mouse anti-rabbit immunoglobulin G conjugated to horseradish peroxidase (Jackson Immunoresearch). Blots were developed using West Pico Chemiluminescent Substrate (Thermo Scientific, Rockford, IL) according to the manufacturer’s instructions and visualized by exposure to X-ray film. All of these experiments were conducted with at least three biological replicates.

For studying intracellular accumulation of CTB subunits in *E coli*, bacteria were grown up to 4 hours following induction by 0.2% of arabinose. Post induction, bacterial cultures were mixed with equal volume of translation-translocation halt cocktail (200 μg/ml chloramphenicol, 200 mM sodium azide, and 9.5% ethanol) [27], and immediately placed in an ice water bath for 15 minutes of incubation to arrest protein synthesis and translocation. Cells were harvested by centrifugation at 14000 rpm for 15 minutes at 4°C. Supernatants were preserved and used for CT-ELISA experiment as described before. Pellets were then suspended in TME buffer (20 mM Tris-Cl pH 8.0, 2mM β-mercaptoethanol, 1 mM EDTA), preferably 1/10^th^ volume of the original culture and disrupted by sonication. After centrifugation at 13,000 rpm at 4°C for 15 min to remove any unbroken cells, crude cell lysates were used for measurement of protein concentration using Bradford assay (BioRad, USA). Equal amount (= />80 μg) of protein samples were mounted on to 15% SDS-PAGE. Blots were probed with classical CTB-specific monoclonal antibody (anti-Cla CTB, 1:5000) or anti-beta lactamase (anti-βla 1:500) antibody. A portion of the same blot was stained with Ponceau S staining solution (Sigma-Aldrich, St Louis, MO, USA) to validate whether equal amount protein samples were transferred in the blot. Blots were developed using West Pico Chemiluminescent Substrate (Thermo Scientific, Rockford, IL) according to the manufacturer’s instructions and visualized in ChemiDoc XRS^+^ system (BioRad, USA). Densitometric analysis of band intensities was performed using the Multi Gauge software V 2.3 (Fuji Film). DOI: http://dx.doi.org/10.17504/protocols.io.bastieen

### RNA isolation and Reverse transcription (RT) PCR

For isolation of total RNA from *V*. *cholerae* strains, overnight grown bacterial cultures were diluted 1:100 in AKI+NaHCO_3_ medium and grown up to six hours under static condition at 37°C. Cells were harvested, and total RNA was extracted using TRIZOL reagent (Invitrogen, USA). Bacterial pellet was suspended in 1 ml of TRIZOL reagent. To this suspension, 200 μl of chloroform was added, mixed well and centrifuged to separate aqueous layer from the organic layer. Total RNA containing aqueous layer was collected and RNA materials were recovered as insoluble pellet by precipitation with 1 volume of isopropanol, washed with 70% ethanol and dissolved in Diethyl pyrocarbonate (DEPC) treated sterile water. Contaminating DNA, if any, present in the preparation was removed by digestion with RNase free DNase I (Thermo Fisher Scientific) for 30 min at 37°C. Isolated RNA samples were then stored in aliquots at –70°C for future use. Following DNaseI treatment, 2μg of total RNA from each samples were used as template for the RT reaction using the RevertAid first strand cDNA synthesis kit (Thermo Fisher Scientific, Waltham, MA, USA). The quantification of target gene by quantitative PCR (qPCR) was performed using 2X SYBR green PCR master mix (Applied Biosystems, USA) and specific primers for each transcript (Table 1) in LightCycler 480 Real-Time PCR System (Roche Applied Science, Penzberg, Germany). Data analysis was performed using the LightCycler 480 software (Roche Applied Science, Penzberg, Germany). The relative expression ratio of the *ctxB* transcript was calculated in comparison to the internal control *recA*.

While using the same protocol described above, total RNA from *E*. *coli* strains was isolated using bacterial cell pellet obtained from 5 ml cultures grown in LB medium supplemented with 0.2% w/v L-(+) arabinose. Semi-quantitative reverse transcriptase (RT) PCR assay was carried out separately using 4 μl of this newly synthesized cDNA and with gene specific primers targeted to *ctxA* and *ctxB* genes. The house keeping gene *16s rRNA* was used as an internal control for normalization of the amplicon intensities among different strains. Amplicons obtained in each of the PCR assay was electrophoresed onto 2% agarose gels and documented (Gel Doc 2000, BioRad, USA). Densitometric analysis of band intensities was performed using the Multi Gauge software V 2.3 (Fuji Film). All of the experiments were performed at least in triplicate.

### *In silico* analysis of CTB signal peptide

To understand whether the H20NCTB_SS_ may affect signal peptide processing proficiency of the type one signal peptidase, identification of the three distinct domains in the CTB signal sequence was the first objective we addressed. Using a five amino acid sliding window, net charge of the peptide at pH 7.8 was calculated using INNOVAGEN Protein Calculator (https://pepcalc.com/protein-calculator.php) and the grand average of hydropathy (GRAVY) score for the respective peptides were calculated using protein GRAVY server (http://www.bioinformatics.org/sms2/protein_gravy.html) by adding the hydropathy values [28] of each amino acid residues and dividing by the number of residues in the sequence or length of the sequence. Increasing positive score indicated greater hydrophobicity. *In silico* secondary structure prediction of WT and H20N CTB signal peptide region was performed through CFSSP (Chou & Fasman Secondary Structure Prediction Server), an online protein secondary structure prediction server (http://www.biogem.org/tool/chou-fasman) which predicts secondary structure of proteins from amino acid sequences using Chou-Fasman algorithm [29].

### Molecular modeling and simulation

Full length structure of the wild type and mutant protein was modeled using MODELLER software where the template structure was selected through PSI BLAST search and the template structure was retrieved from PDB database (PDB ID 1FGB). Apart from MODELLER, alternative modeling tools such as RaptorX [30] and I-TASSER [31] were also used. RaptorX server predicts 3D structures for protein sequences without close homologs in the Protein Data Bank (PDB). For any given protein sequence, RaptorX predicts its secondary and tertiary structures, contacts, solvent accessibility, disordered regions, and binding sites. It also provides P-value for model quality assessment. I-TASSER which stands for Iterative Threading ASSEmbly Refinement is a tool for predicting the 3D structure model of protein from amino acid sequences. It uses fold recognition or threading techniques to identify templates from the PDB. In the next step by using replica exchange Monte Carlo simulations, it reassembles structural fragments detected by the threading method into a full-length 3D protein model. It also performs *ab initio* modeling of the protein sequence for which there is no template available. Besides protein modeling, I-TASSER also predicts potential ligand-binding sites, gene ontology and enzyme commission using structural similarity to the known proteins in protein function databases.

The template structure was devoid of the signal sequence part. So, to get the approximately stable conformation of CTB protein along with its WT and H20N signal sequence, we used Gromacs 4.5 software to run 10 nanoseconds (ns) molecular dynamics simulation in solution. The simulation parameters were used as described [32]. The protein-peptide interactions were studied in ClusPro 2.0 server. Structural analysis and visualization was performed in PyMol software.

### Accession numbers

Nucleotide sequences of the *ctxA*, *ctxB1* and *ctxB7* genes cloned in pBAD plasmids were deposited in to GenBank under the accession numbers MN829555, MN833233 and MN833234, respectively. Amino acid and DNA sequence of the CTB K3E mutant is available in GenBank under the accession number MN864751. Nucleotide sequences of the *ctxB* locus from *V*. *cholerae* O1 strains are available in GenBank with the accession numbers MN833235 (L4867), MN833236 (IDH00161), MN833237 (IDH00790), MN833239 (IDH00990), MN833240 (IDH03454), MN833241 (IDH03595) and MN864752 (L19494). DNA sequence of CTX promoter regions from *V*. *cholerae* strains are available in GenBank under accession numbers MN829553 (IDH03371), MN829554 (IDH03595) KJ647311.1 (IDH00990), MN864753 (IDH00161), MN864754 (IDH00790) MN864755 (L4867) and MN864756 (IDH03454).

## Results

### Overproduction of classical CT by Haitian variants of *V*. *cholerae* O1 strains isolated from Kolkata under AKI-inducing conditions

**Fig 1:**
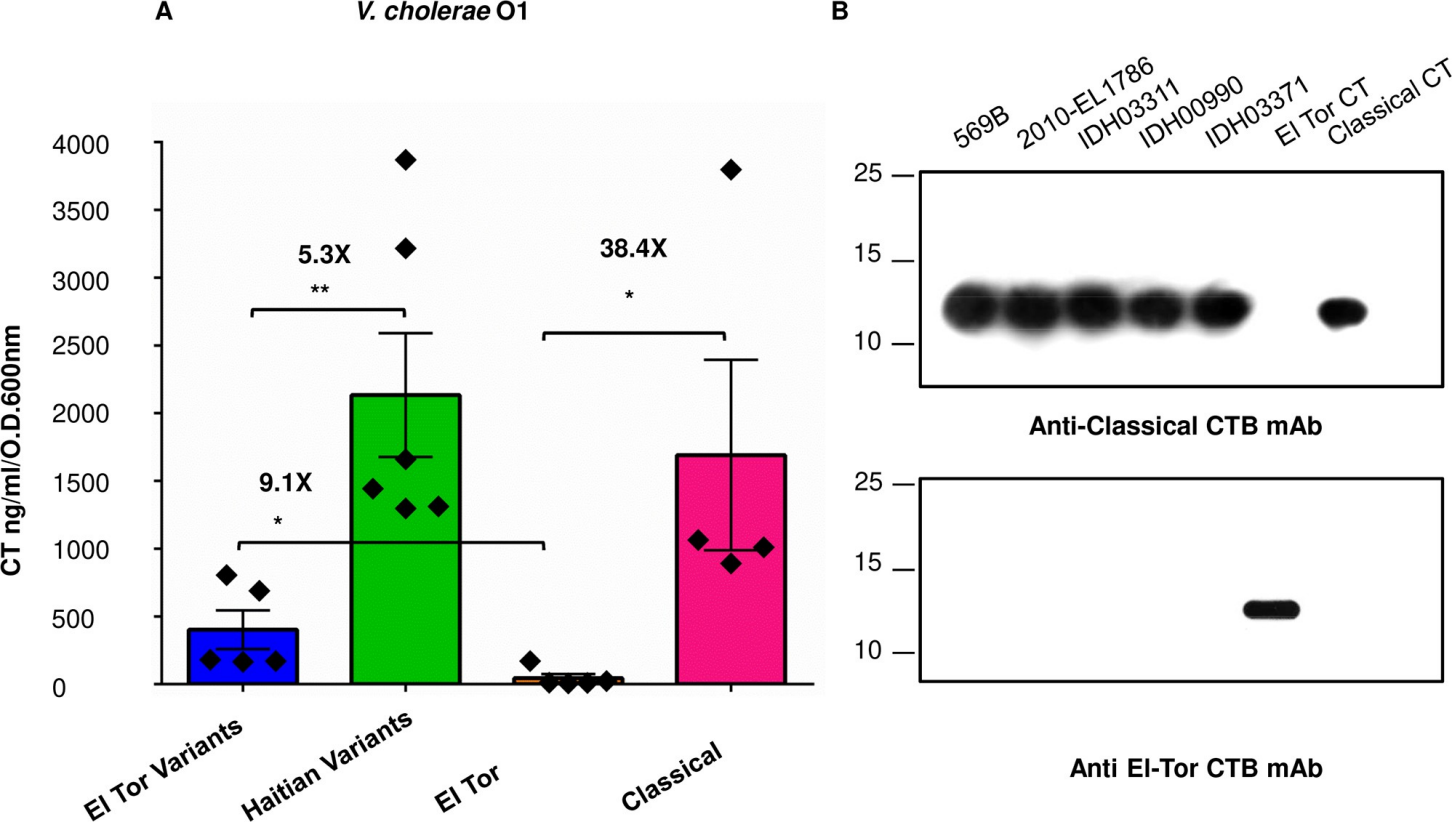
Study of toxin secretion in *V*. *cholerae*. (**A**) Quantification of total cholera toxin (CT) production by *V*. *cholerae* O1 strains isolated from Kolkata under AKI conditions. CT (ng/ml/O.D.600nm) produced by *V*. *cholerae* O1 Haitian variants L19494 (2006), IDH00990 (2008), IDH03311 (2010), IDH03454 (2011), IDH03595 (2011), and the Haitian outbreak isolate 2010EL-1786 was measured and compared with that secreted by El Tor variant strains K3314 (2005), L4867 (2006), IDH00161 (2007), IDH00790 (2008), and IDH03371 (2010), El Tor strains V24, V7, V32, V54 and V100, and classical strains L362, GP15, GP145, GP147, [20]. *In vitro* CT production was determined by GM_1_ CT ELISA as described in materials and methods. Every black diamond represents a single strain. The mean value of at least three individual experiments for each strain is shown with error bars signifying standard errors for each group. An unpaired two-tailed student’s t test was used to analyse the statistical significance of the data. (* *P* value <0.05, **P value <0.005). “X” indicates the fold difference in values. (**B**) Western immunoblot results of the culture supernatants of representative *V*. *cholerae* O1 strains. 20 ng of the purified classical CT was used as positive control for immunoblotting with the monoclonal antibody against classical CTB.

To examine if the Haitian variants could produce higher amount of CT than contemporary El Tor variant strains, cell-free culture supernatants of twenty *V*. *cholerae* O1 strains were used to determine the amount of CT produced under AKI conditions. We found that the entire lot of Haitian variant strains secreted significantly higher amount of CT in comparison with the El Tor variant strains isolated during the same period (Fig 1, *P* value <0.005 as calculated through in GraphPad PRISM (V.6.0). Two out of the six Haitian variants produced CT more than 3000 ng/ml/O.D.600nm. The Haitian variant strain IDH03311 which was isolated in 2010, produced the largest amounts of toxin (~3870 ng/ml/O.D.600nm). Two (IDH00790, IDH03371) out of the five El Tor variant strains secreted CT more than 500 ng/ml/O.D.600nm whereas the remaining three secreted below 200 ng/ml/O.D.600nm. As expected, all of the classical strains produced much larger amount of CT than the El Tor strains matching closely the amount produced by the Haitian variants (*P* value <0.05). Except GP145, which secreted the lowest amount of CT (~891 ng/ml/O.D.600nm) among the four classical strains, all of the three remaining produced CT more than 1000 ng/ml/O.D.600nm while the classical strain L362 produced ~3797 ng/ml/O.D.600nm of CT. As reported earlier [20], all of the El Tor strains secreted extremely low amount of CT while grown under identical conditions. Except V100, which produced the highest amount of CT (~170 ng/ml/O.D.600nm) among the El Tor strains, all of the four El Tor strains secreted less than 50 ng/ml/O.D.600nm of CT while V32 produced the lowest amount of toxin (6 ng/ml/O.D.600nm) examined in the study. The Haitian outbreak isolate 2010EL-1786 produced 1659 ng/ml/OD600nm of CT. It is to be mentioned that at least four individual colonies of every single strain were picked up for the ELISA assays. Although variations were observed between individual replicates (between four colonies of a single strain), cumulative toxin production data supported the aforesaid observation.

Western blot with immunoprecipitated culture supernatants using a classical CTB-specific monoclonal antibody showed that indeed all of the tested strains including the classical control strain 569B and the Haitian isolate 2010EL-1786 secreted identical CTB epitype (CTB1) (Fig 1B). These results are consistent with previous reports that Haitian variant isolates produce excess cholera toxin [4,14].

**Fig 2:**
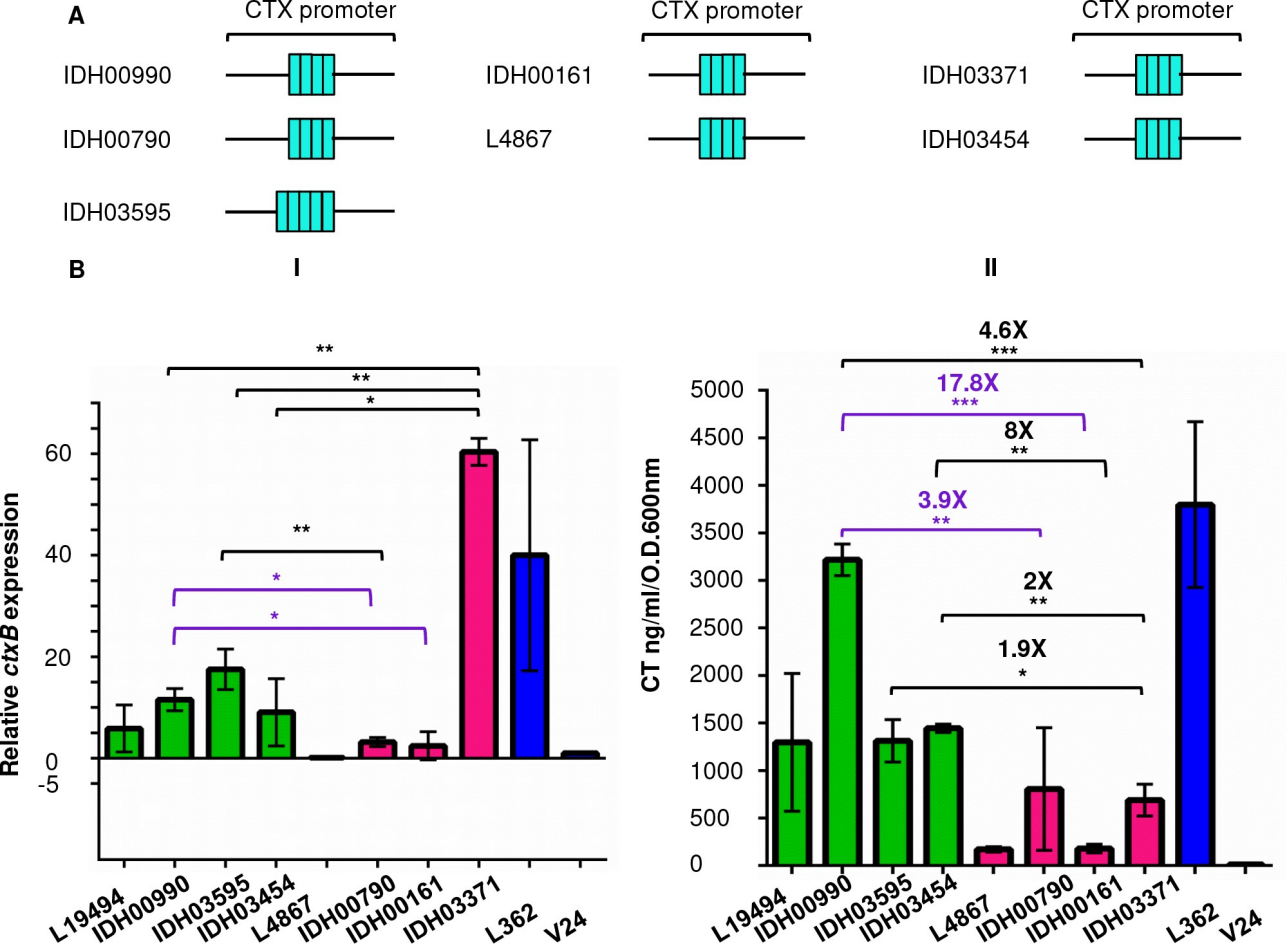
Analysis of the role of transcriptional regulation in CT overproduction among Haitian variants. **(A**) Schematic representations of the CTX promoter region in *V*. *choleae* isolates. Turquoise rectangles each represent a single 5′-TTTTGAT-3′ heptad repeat. (**B**) qRT PCR (I) and measurement of CT production (II). *ctxB* expression was normalized to the *recA* gene with the expression of the *ctxB* in V24 set to 1.0. Results are from two independent experiments performed in triplicate. Purple brackets identify two separate cases where transcriptional upregulation of the *ctxB* gene might have resulted in the higher production of CT under *in vitro* condition. Error bars represent standard deviations from at least two biological replicates. Statistical significance is indicated at *P* values (* P value <0.05, **P value <0.005, ***P value <0.0005).

The CTX promoter contains a high AT rich region composed of tandem repeats of the sequence TTTTGAT. Previously these heptad repeats have been shown as binding sites for the transcriptional regulators ToxT and H-NS [33–34]. We speculated if the heightened CT production may simply result from elevated *ctxB* gene expression and whether heptad variations, if present in the currently analysed set of isolates may play a part in this. We found that all of the strains had four TTTTGAT heptad repeats except IDH03595 (GenBank accession number: MN829554) which had an additional repeat (Fig 2A). To verify whether transcriptional regulation could also play a role in CTX overproduction in the Haitian variants, we evaluated the relative expression of *ctxB* in ten *V*. *cholerae* isolates. Quantitative PCR (qPCR) analysis showed that while the El Tor variant strain IDH03371, a 2010 isolate showed highest fold change in *ctxB* transcript level (~60 fold) relative to *ctxB* expression in the El Tor strain V24, it produced ~689 ng/ml/O.D.600nm of CT which was lower than three out of the four Haitian variant strain tested (Fig 2B I). On the other hand, Haitian variant strains IDH00990, IDH03454 and IDH03595 showed significantly lower expression of *ctxB* mRNA in comparison with IDH03371 (*P* value <0.005, <0.05 and <0.005 respectively). Nevertheless, all of them produced at least 2 times higher CT than the 2010 El Tor variant strain. Interestingly, difference in CT production between another Haitian variant strain IDH03595 (which has 5 TTTTGAT heptad repeats in the CTX promoter region) and the El Tor variant IDH00790 (4 TTTTGAT repeats in the CTX promoter) remained non-significant although the former showed significantly higher abundance of *ctxB* mRNA (*P* value <0.005), suggesting that the additional TTTTGAT heptad sequence might have a role in transcriptional upregulation of the *ctxAB* operon in this strain. IDH00990 produced highly increased amount of CT than IDH00790 (*P* value <0.005), and IDH00161; a 2007 El Tor variant isolate (*P* value <0.0005) possibly due to the relative higher amount of *ctxB* transcript level (*P* value <0.05). Taken together, qPCR analysis did not establish any connection between a possible role of transcriptional regulation and the overproduction of CT in the Haitian variants. It was also observed that the number of repeat did not correlate with the change in relative *ctxB* mRNA level (Fig 2B I) or the amount of toxin produced *in vitro* (Fig 2B II).

### *ctxB7* contributes to higher amount toxin production

**Fig 3:**
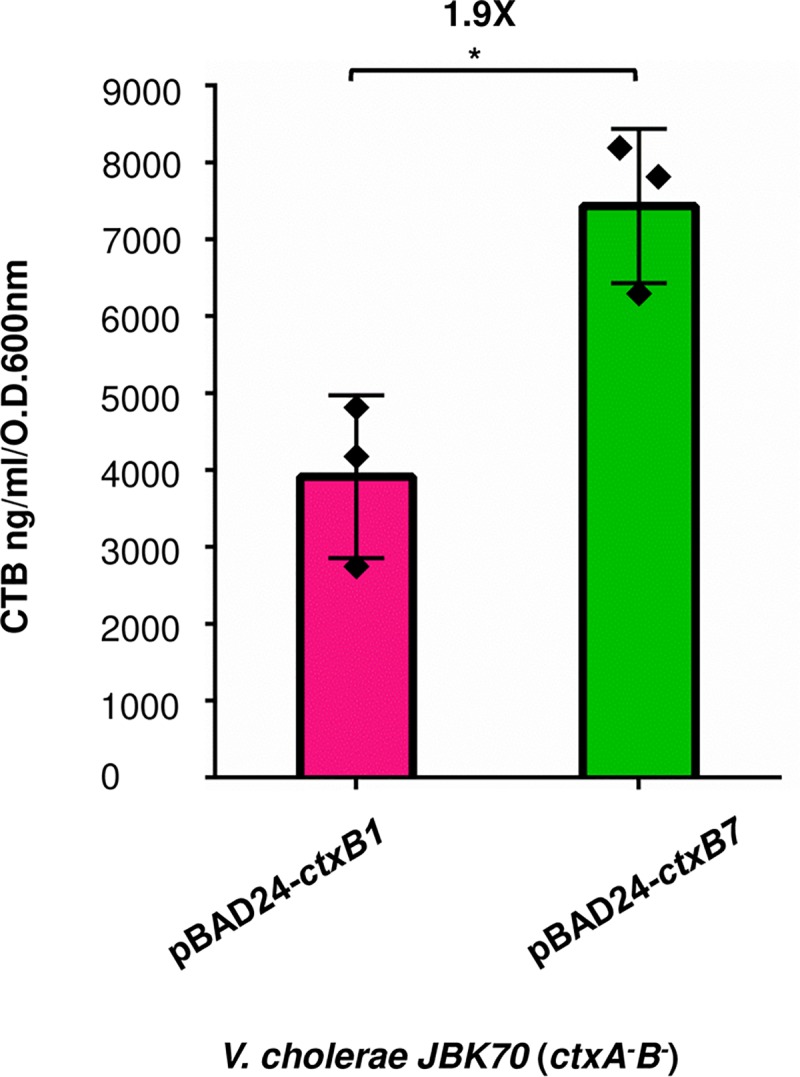
Measurement of toxin subunit secretion by recombinant *V*. *cholerae* cells. CT ELISA with culture supernatants of recombinant JBK70 strains expressing either *ctxB1* or *ctxB7* from pBAD24 (See Table 1). CT production was determined by GM_1_ CT ELISA in triplicate. Mean value of at least three individual experiments (represented by black diamonds) is presented. Standard deviations are indicated with error bars. Unpaired two-tailed student’s t test was used to analyse the statistical significance of the data. (*P value <0.05). The fold difference in CT values (ng/ml/O.D.600nm) is presented by “X”.

To understand whether the *ctxB7* allele itself may play a part in higher CT production by Haitian variant strains, *V*. *cholerae* JBK70 (an isogenic Δ*ctxAB* mutant of N16961) harboring the arabinose inducible vector pBAD24 alone (AN1), pBAD24 with *ctxB1* (AN2) or *ctxB7* (AN3) were examined for their ability to produce CTB. This allowed us to directly compare the secretion of CTB to supernatant in the same underlying genomic context (*hns* and *vieA* unchanged). Upon arabinose induction, amount of CTB was found to be significantly higher (7432 ng/ml/O.D.600nm) in the supernatant of the strain AN3 compared to that of the strain AN2 (3912 ng/ml/O.D.600nm) (Fig 3, *P* value 0.034; two-tailed standard t test). Interestingly, JBK70 strain expressing a signal sequence mutant of CTB, which has a neutral N terminus (K3E) showed markedly defective CTB production (~321 ng/ml/O.D.600nm, *P* Value <0.002, S1 Fig). Both of the strains produced barely detectable CTB under repressing condition.

**Fig 4:**
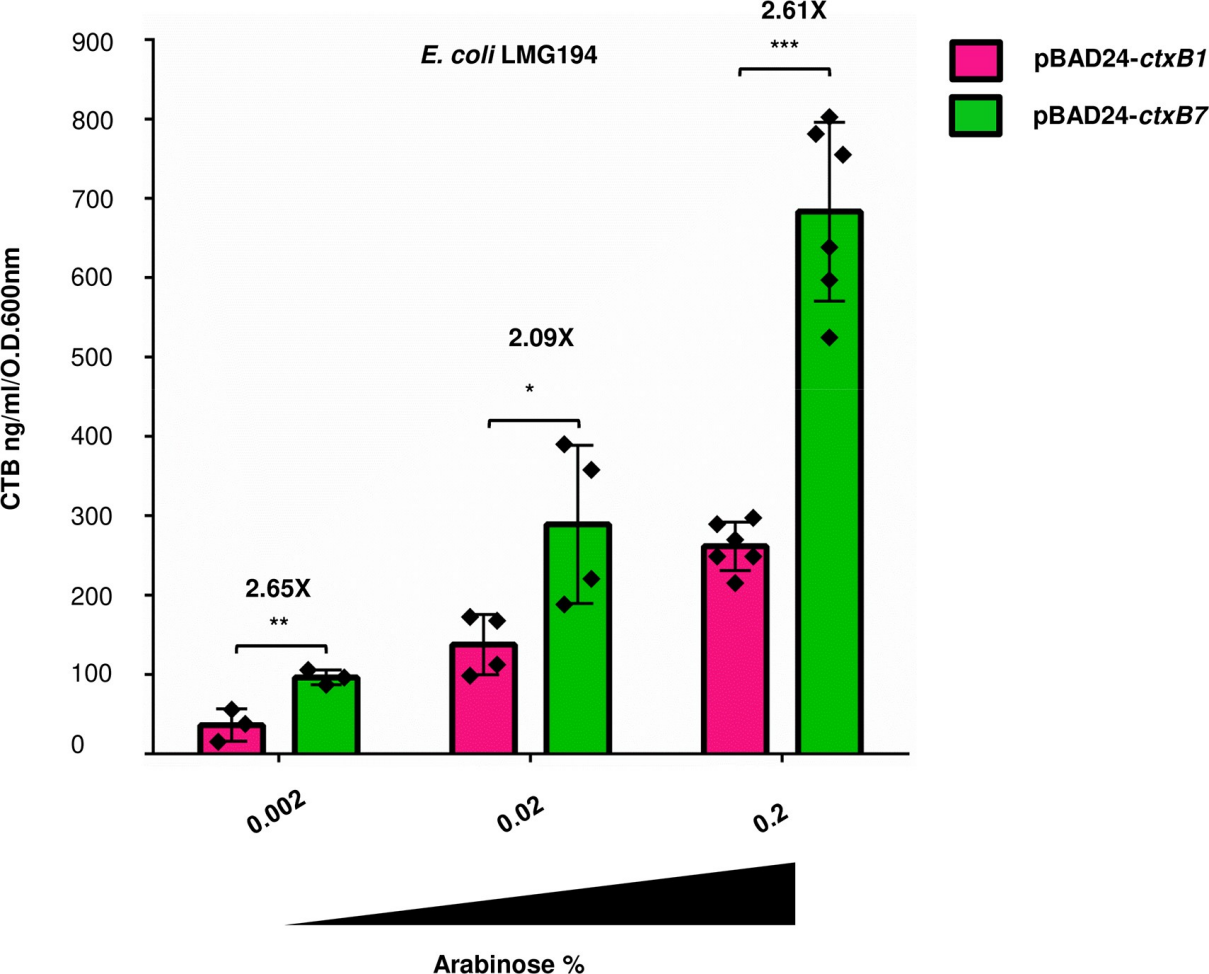
CTB Secretion profile of *E coli* strains. AN5 (LMG194/pBAD24-*ctxB1*) and AN6 (LMG194/pBAD24-*ctxB7*). Expression of *ctxB* gene was induced from pBAD promoter by the adding 0.002, 0.02 or 0.2% of arabinose in the media and secretion of CTB was measured from cell free culture supernatants of the bacterial strains. Individual experimental data has been represented by black diamonds. For each set of samples, mean values were plotted. Error bars indicate standard deviations of at least three individual experiments. Statistical significance of the data was calculated using Two-way ANOVA. (* *P* value <0.05, **P value <0.005, ***P value <0.0005). See text for further details.

To rule out the possibility that enhanced production of CTB by AN3 could be due to the influence of any ‘unknown’ *V*. *cholerae* specific factor, the same recombinant plasmids were expressed under identical conditions in the *E*. *coli* K12 strain LMG194. Measurement of CTB production in culture supernatants of arabinose induced AN5 (LMG194/pBAD24-*ctxB1*) and AN6 (LMG194/pBAD24-*ctxB7*) showed similar results where AN6 accumulated more than 2.5 fold higher CTB (~685 ng/ml/O.D._600nm_) than the former which produced 261 ng/ml/O.D._600nm_ of CTB (Fig 4, *P* value <0.0001). Furthermore, it was also seen that although the overall level of CTB dropped in both the strains with the lowering of arabinose concentration 10 to100 fold down to 0.002%, the fold difference in toxin subunit secretion remained nearly the same. However, the levels of accumulated toxin subunits in cell free media fractions in both of the cases were more than 10 fold lower what was observed in the JBK70 background. This difference in CT secretion between *V*. *cholerae* and *E coli* was not unexpected as expression of cholera toxin genes in *E*. *coli* results in the accumulation of cell-associated cholera toxin [35]. It is to be noted that our observation did not rule out the possibility of cell lysis and thereby release of CTB subunits accumulated inside the cells in to the media fraction. Since routine variations including "intraassay" (within the plate, replicate values),"interassay" (between plates) and "day to day" were recorded among ELISA standard curves and sample values, at least four biological replicates (for each of the tested strains) with most reproducible data sets were selected for the analysis.

### Expression of *ctxB7* results in higher production of cell associated enterotoxin in *E coli*:

**Fig 5:**
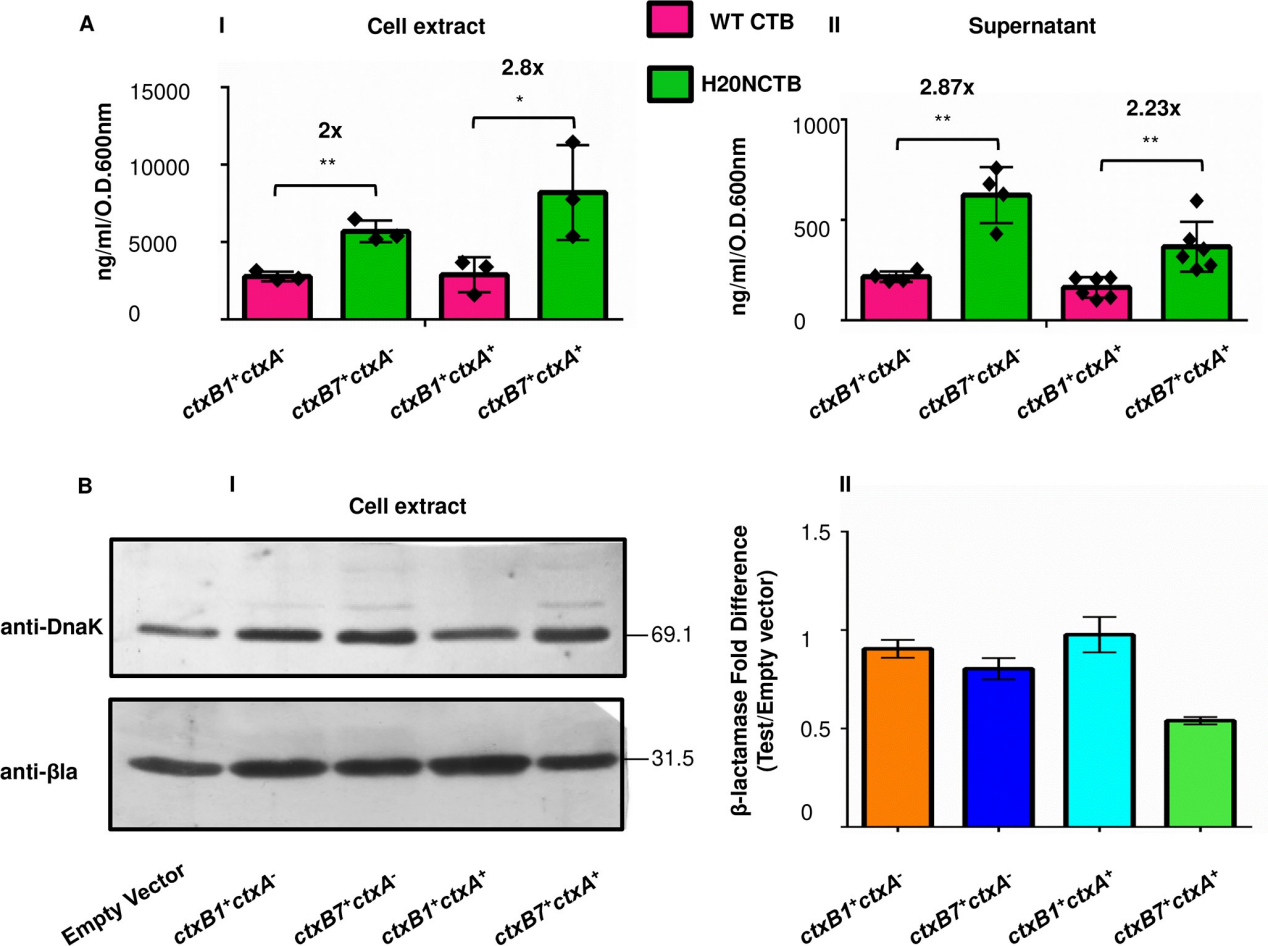
*ctxB7* expression results in higher accumulation of cell bound enterotoxin in *E*. *coli*. (**A**) Determination of toxin subunit accumulation in the cell extract (**I**) and culture supernatant (**II**) of *E coli* strains AN8 (LMG194/pBAD24-*ctxB1*/pBAD33), AN9 (LMG194/pBAD24-*ctxB7*/pBAD33), AN10 (LMG194/pBAD24-*ctxB1*/pBAD33-*ctxA*) and AN11 (LMG194/pBAD24-*ctxB7*/pBAD33-*ctxA*). Co-expression of *ctxB1/ ctxB7* and *ctxA* genes was induced from pBAD promoter by adding 0.2% of arabinose in the media. Accumulation of CT or CTB was measured from cell extracts or cell free media fractions of bacterial strains producing either pre-WT CTB (dark pink) or pre-H20N CTB (green) inside the cell. Mean values of at least three independent experiments (each experiment has been represented by black diamond) were plotted for each set of samples. Error bars indicate standard deviations. Statistical significance of the data was calculated using Two-way ANOVA. (* *P* value <0.05, **P value <0.005). (**B**) **I**. Western immunoblot for quantifying efficient lysis of bacterial cells. DnaK was used as the loading control for normalization to equal amount of total protein in each sample. **II**. Bands were quantitated using MultiGuage V2.3 image analysis software. See text for further details.

Previous studies showed that the putative extracellular transport signal of CT is located on its B subunit [36]. We therefore reasoned that elevated levels of CTB accumulation in supernatants of *V*. *cholerae* and *E coli* cells synthesizing pre-H20N CTB from *ctxB7* allele may also affect CT secretion. To determine the effect of *ctxB7* in holotoxin production, *ctxA* gene from *V*. *cholerae*, which codes for cholera toxin A subunit (CTA) was expressed under the pBAD promoter of another arabinose inducible expression vector, pBAD33 (Table 1) along with pBAD-CTB in *E coli* strains AN10 (AN5:CTA) and AN11 (AN6:CTA). Strains AN8 (AN5/pBAD33) and AN9 (AN6/pBAD33, Table 1) were used as vector controls for pBAD33 which produced CTB tagged with WTCTB_SS_ and H20NCTB_SS_ from *ctxB1* and *ctxB7*, respectively. Measurement of accumulated toxins with the culture supernatants and cell extracts of each of the four strains showed greater abundance of cell associated toxin subunits in AN9 and AN11 compared to AN8 and AN10, respectively (Fig 5A I). It could be seen that strain AN11 produced (~8197 ng/ml/O.D.600nm, *P* value 0.04) in the cell associated form which was 2.8 fold higher than the amount of accumulated CT by strain AN10 (~2891 ng/ml/O.D.600nm). Although the amounts of CT released from the two bacteria dropped sharply while compared to that retained in the cell extracts, nevertheless 2.23 fold differences between the CT levels measured *in vitro* remained extremely significant (*P* value <0.005). The ancestral strains AN8 and AN9 showed near similar CTB accumulation profile. AN9 accumulated 2 fold more toxin subunits inside the cell compared to AN8 (*P* value <0.005) which produced CTB ~2781 ng/ml/O.D.600nm. Western blot analysis with anti-beta lactamase antibody confirmed efficient lysis of all the bacterial strains during sample processing (Fig 5B), thereby ruling out any possible consequence because of uneven cell lysis in the release of the toxin subunits.

To study whether the increase in the mature CTB pool was due to efficient pre-H20N CTB processing or simply resulted from increased CTB production from the *ctxB7*, intracellular CTB level inside bacterial strains expressing either *ctxB1* or *ctxB7* was carefully monitored. Following induction with 0.2% arabinose, bacterial cultures were mixed with equal volume of translation-translocation halt cocktail. Cell extracts were processed for western blot as described in “Methods”.

**Fig 6:**
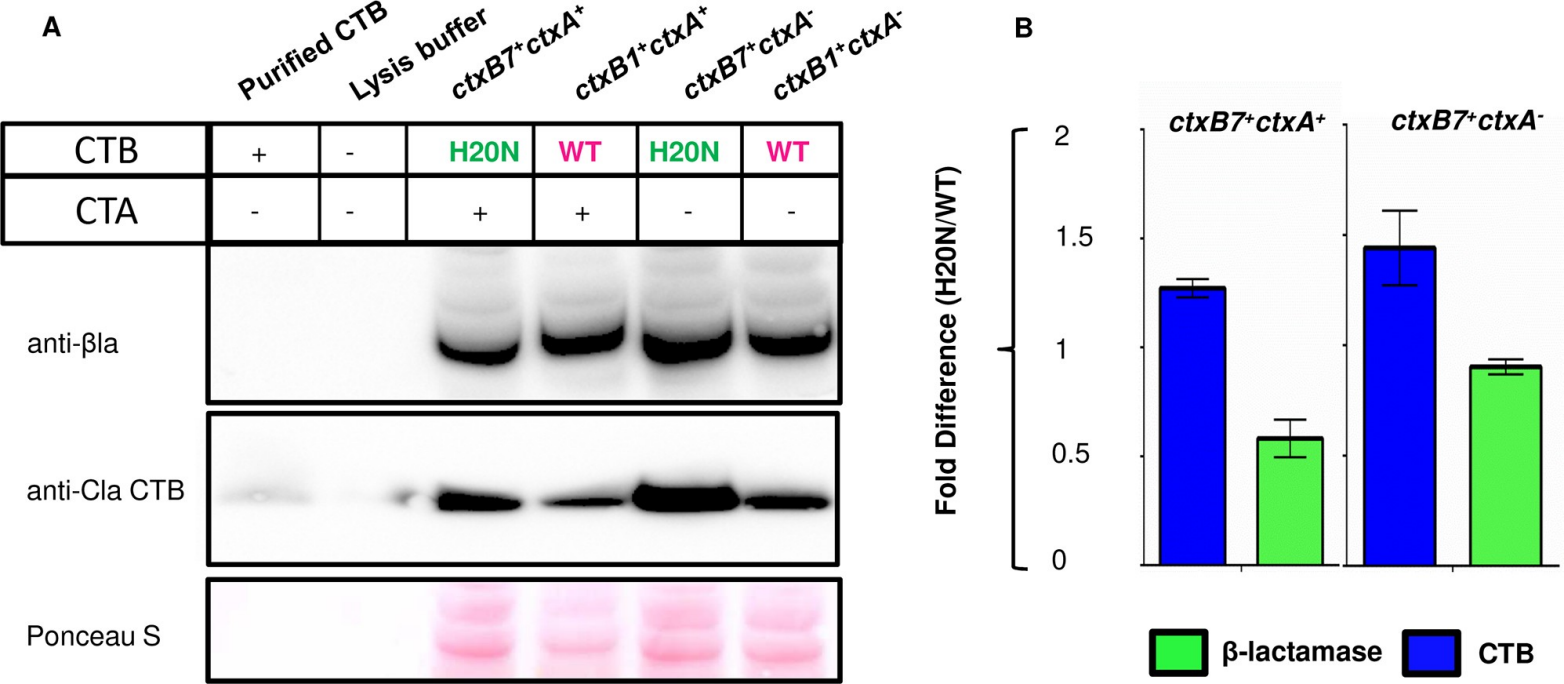
Cellular abundance of mature H20N CTB subunit results in increased toxin production by *E coli* strains with *ctxB7* allele. (A) The abundance of mature H20N CTB (relative to mature WT CTB) is shown in *E*. *coli* AN9 (*ctxB7*^+^*ctxA*^-^) and AN11 (*ctxB7*^+^*ctxA*^+^) in comparison with AN8 (*ctxB1*^+^*ctxA*^-^) and AN10 (*ctxB1*^+^*ctxA*^+^) strains. Following arabinose induction, samples were processed for western blot as described under “Methods”. A Ponceau S stained portion of the membrane was used as a loading control to normalize for equal amounts of total protein on the blot. 20 ng of the purified classical CT was used as positive control for immunoblotting. Cell lysis was quantified by using antibody against β-lactamase. (B) Fold difference in band intensity relative to WT (H20N/WT) was quantitated using MultiGuage V2.3 image analysis software.

Western blot experiment with anti-CTB mAb showed higher accumulation of mature CTB subunits inside the cells expressing H20N CTB from the *ctxB7* allele (Fig 6A). Densitometric analysis showed 1.4 fold difference in the band intensity of H20N CTB in comparison with WT (Fig 6B). It is to be noted that chloramphenicol in the translation-translocation halt cocktail inhibited protein synthesis, whereas translocation of pre-proteins was arrested by sodium azide, which is an extremely potent inhibitor of protein export *in vivo* [37]. Unlike the previous experiments, this modification in sample preparation thus eliminated any possible interference of background CTB translation and translocation during sample preparation, by freezing all of the cellular proteins at a given time. This result further supported our previous observation where our CT ELISA data showed that *E*. *coli* stains secreted heightened amount of toxin subunits from the *ctxB7* allele, no matter whether the CTA subunit was also produced alongside CTB or not (Fig 5A II). Quantitation of cell lysis among the tested strains showed that *E coli* strains expressing *ctxB7* lysed rather poorly (Fig 6B). This observation further supported our perception that the difference in detectable CT level between the strains was not due to uneven cell lysis during sample preparation for the assays. Although, it remained undetermined whether the observed increase in the mature CTB pool was due to efficient pre-H20N CTB processing by the signal peptide or simply resulted from increased CTB production the *ctxB7*, as we could not detect unprocessed precursor fraction in case of *E coli* cells producing either of the pre-WTCTB or pre-H20N CTB.

**Fig 7:**
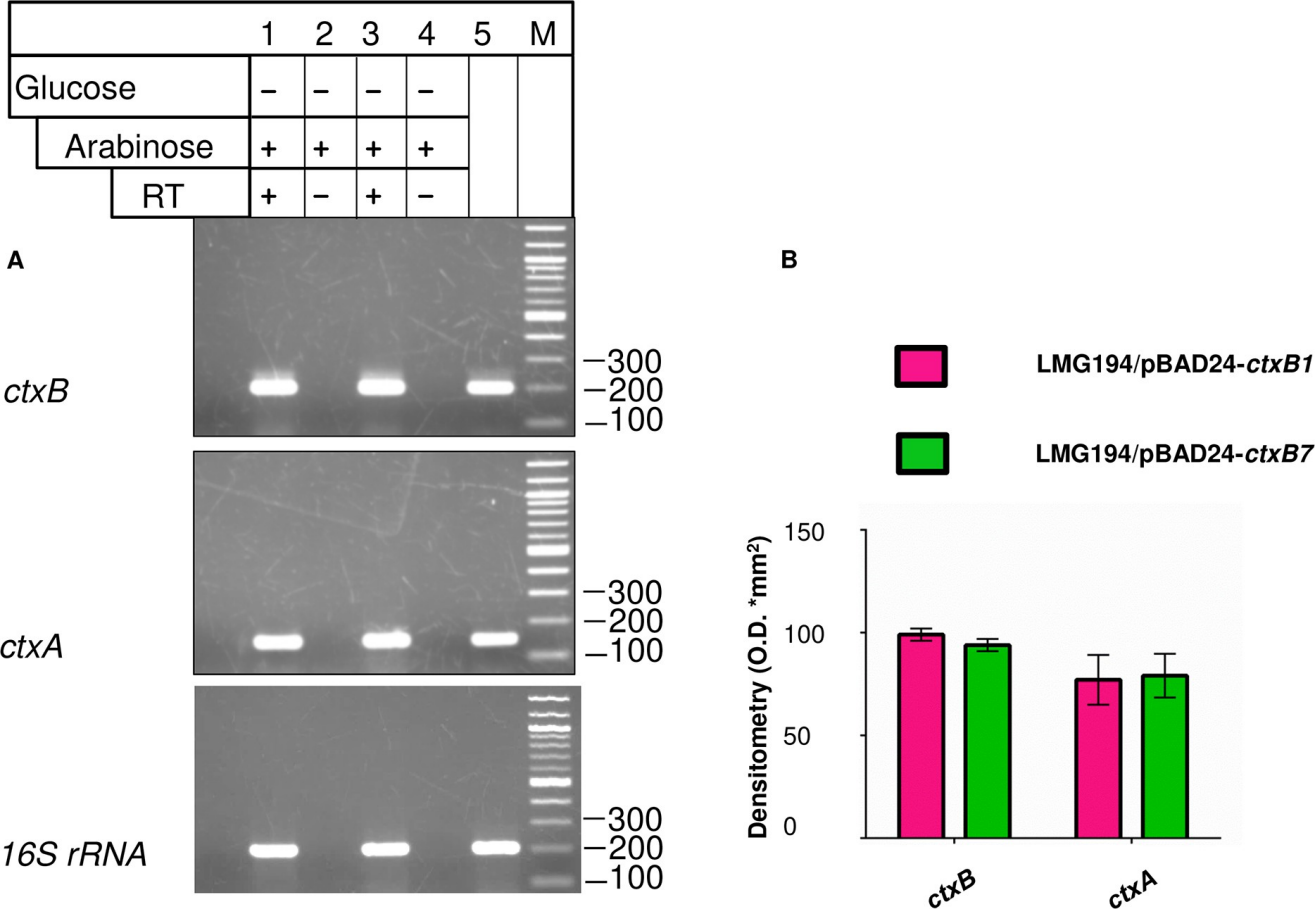
Semi quantitative measurement of *ctx* mRNAs produced upon arabinose induction. (**A**) Total cellular RNA extracted from *E coli* strains AN10 and AN11 grown in presence of 0.2% arabinose was used for Reverse transcriptase (RT) PCR. Lane 1 and 3: cDNA samples from AN10 and AN11 PCR amplified with gene specific primers. Lane 2 and 4: RT negative control for total cellular RNA isolated from AN10 and AN11, respectively. Lane 5: PCR amplified products from the genomic DNA of *V*. *cholerae* and *E coli* as positive controls. *16S rRNA* gene was used as the housekeeping control. (**B**) Densitometric analysis of band intensities from Fig 7A using the Multi Gauge software V 2.3 (Fuji Film). All of the experiments were performed at least in triplicate. Error bars represent standard deviations of a minimum of three individual experiments.

We were unable to observe any noteworthy difference in either of the *ctxB1* or *ctxB7* transcript levels in AN10 or AN11 (Fig 7). This observation allowed us to conclude that higher production of CT by AN11 compared to AN10 seemed to indicate that both in *V*. *cholerae* and *E*. *coli*, *ctxB7* promoted increased CT production possibly through a post-transcriptional mechanism.

### *A* structural alteration in the H20NCTB_SS_ and its possible involvement in pre CTB processing

Motivated by our toxin production data, which showed higher amount of toxin production by recombinant *V*. *cholerae* and *E coli* strains expressing pre-H20N CTB from the *ctxB7* allele, we decided to characterize the CTB signal peptide structure to see whether the H20N substitution had any possible effect on the elevated accumulation of toxin subunits in bacterial cell extracts or cell free culture supernatants. Therefore, identification of three distinct domains [38] in the CTB signal sequence was the initial objective that we addressed. The overall net charge of the CTB signal peptide was found to be +2 due to the presence of two lysine residues at positions 3 and 5 in the amino (N) terminus. In this study, we also investigated the hydrophilic segment of the CTB signal peptide. Markedly defective protein secretion was observed in the AN12 strain (~ 12 fold defect, *P* value<0.005, S1 Fig) which had a neutral N-terminus due to the substitution of a lysine residue by glutamic acid (K3E). Our results support previous reports where requirement of a net positive charge at the N-terminus was demonstrated for optimal precursor processing and translocation [39]. Following the hydrophilic N terminus, the middle portion (residues 6–15) is rich in hydrophobic amino acids like phenylalanine (F6, F9, and F10), valine (V8 and V12) and leucine (L13 and L14). The hydrophobicity dropped down sharply just before the C region of the signal peptide which consists of 6 residues (S2 Fig).

It was found that two small, neutral amino acids glycine (G21) and alanine A19 are present in -1 and -3 positions whereas large polar residues like histidine or asparagine (H20 or N20 in WTCTB_SS_ or H20NCTB_SS_ respectively) are located at the—2 position counting from the cleavage site.

**Fig 8:**
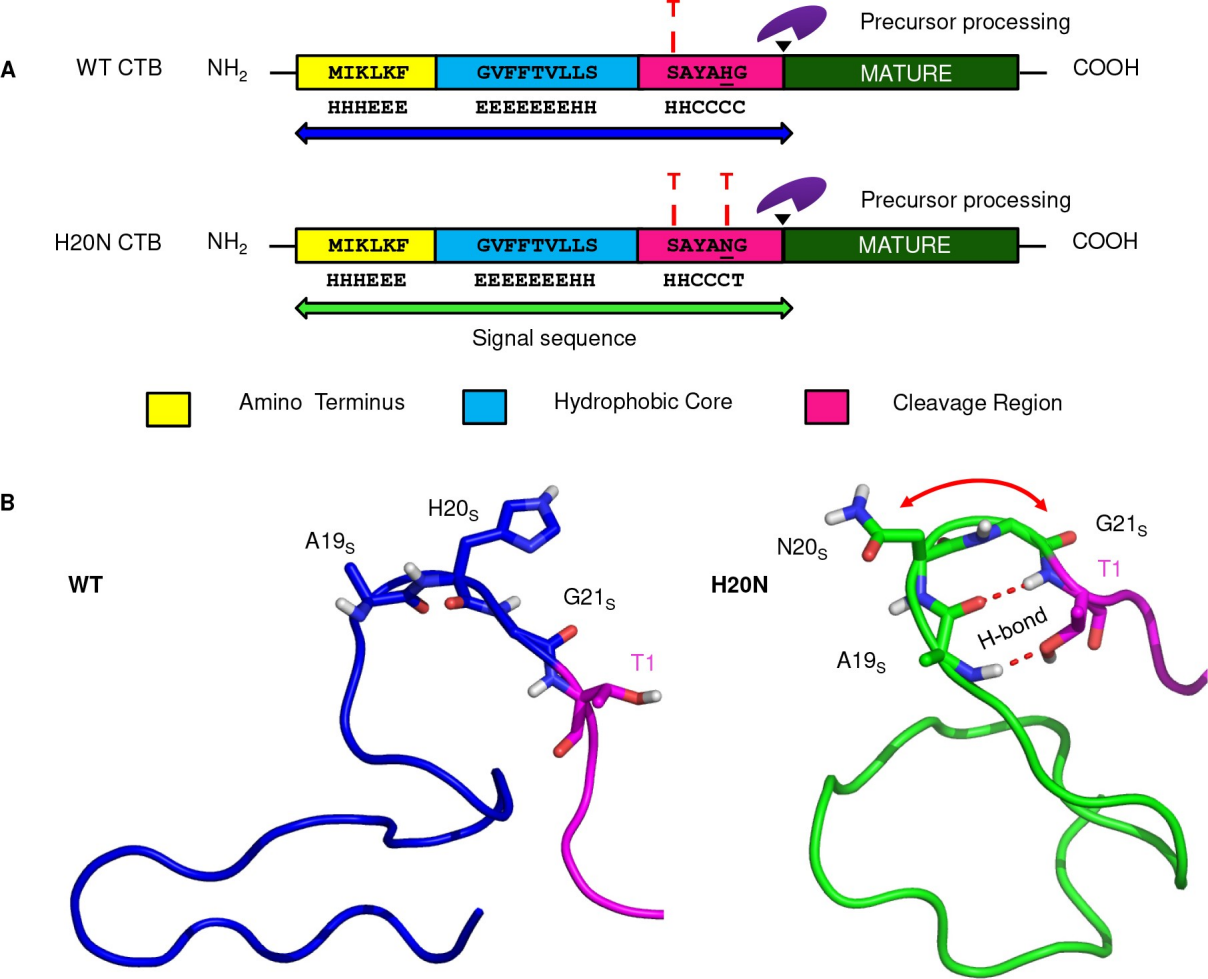
Distinct properties of the H20N CTB signal peptide. (**A**) Schematic representation of the signal sequence of *V*. *cholerae* cholera toxin subunit B protein from O395 and 2010 El-1786. Three different regions in the signal peptide have been shown in three different colors. The N terminal region which bears a net positive charge of +2, has been highlighted in yellow. The hydrophobic core and cleavage regions are shown in cyan and magenta, respectively. Mature portions of the CTB monomers are shown in dark green. The black arrow indicates signal sequence processing site. Histidine and asparagine residues at the -2 site have been underlined. Secondary structure prediction of the WT and H20N CTB signal sequences using Chou and Fasman Secondary Structure Prediction Server (CFSSP) shows diversity in the secondary structures of both WT and H20N CTB signal peptides. Helix (H), Sheets (E), Coils (C) and Turns (T) of both the WT and H20N signal sequences have been indicated over the specific amino acid residues. Position of β turns have been indicated over the specific residues. (**B**) Minimum energy conformation of the WT and H20N CTB resulted from the 10 nano seconds molecular dynamics simulation, showing the conformational change of the H20N CTB signal sequence with respect to WT signal sequence by an introduction of β-turn (indicated by the red curved arrow) between residues A19, N20, G21 and T1.

Based on the overall analysis of the CTB signal peptide, the schematic presentation of three distinct segments in the H20NCTB_SS_ has been proposed (Fig 8A). Previously, the presence of β-turns near the processing site in signal sequences has been reported to play a crucial role in precursor processing and protein secretion [40–41]. Secondary structure prediction by Chou-Fasman algorithm showed introduction of an additional β-turn, across the cleavage region of H20N signal sequence in contrast to the WT, which has only one single turn at the core-cleavage region boundary (Fig 8A). Minimum energy conformation of the H20N CTB showed that H20N mutation incorporated a β-turn in H20NCTB_SS_ through a H-bond interactions between alanine at 19^th^ position (A19) and glycine at 21^st^ position (G21) and also between A19 and T1^M^ (the first amino acid in mature CTB) which was not seen in the WTCTB_SS_ (Fig 8B)_._ Our analysis, therefore suggested the possibility of more efficient precursor processing by the signal peptidase enzyme which could in turn lead to higher CT production by the Haitian variants.

Near identical results were obtained while using different modelling approaches (S3 Fig). From the two structures resulting from RaptorX (S3A and S3B Fig), it became evident that the signal peptide part of the WT structure was quite linear while the H20N structure was making some turns. In this modeled structure, N20 residue was not associated with any H-bonding with the neighboring residues but a slight conformational change could form turns through stable electrostatic interactions. This assumption was true for the H20N structure resulting from I-TASSER (S3C Fig) where N20 was found capable of making H-bonding with A19 and Y18 (represented by red dotted lines), while the WT structure from I-TASSER did not form any turn but involved in a helix formation (S3D Fig).

## Discussion

In recent decades, cholera cases have been increased globally. The 2010 cholera outbreak in Haiti where cholera was never reported earlier drew global attention. The genome wide single nucleotide polymorphism (SNP) analysis of *V*. *cholerae* strains isolated from the Haitian outbreak [12] provided researchers with an opportunity to study the functional significance of unique mutations present in the Haitian strains; *tcpA* allele (*tcpA^CIRS^*), *rtxA* null mutation and *ctxB7* [6].

Our earlier study showed that *V*. *cholerae* O1 strains with *ctxB*7 first appeared in Kolkata during April 2006 and by 2011 and over 90% of the strains carried the signature *ctxB* allele [7]. In a previous report [4], Haitian outbreak isolate was found to exhibit characteristics of hypervirulence, with increased toxin production and hemolysin secretion. Increased pathogenic potential was directly demonstrated as the hypervirulent strain outcompeted other strains in cocolonization studies. Furthermore, one of the recent reports from our group has also shown that Haitian variant strains of *Vibrio cholerae* O1 displayed higher virulence in animal models [17]. However, it was not previously known whether the H20N change in *ctxB7* may have contributed to hypertoxigenic phenotype of the Haiti variant isolates.

This study was undertaken to examine any possible role of *ctxB7* allele in the hypertoxigenic phenotype of Haitian variant strains. Our results showed that the Haitian variants isolated through 2006–2011 produced higher levels of CT compared to the El Tor variants isolated in the same period and could possibly be a factor in their rapid dissemination over time through positive selection for increased virulence. Production of CT is controlled through the transcriptional regulation of the *ctxAB* operon in *V*. *cholerae*. In absence of the virulence activator ToxT [42], *ctxAB* promoter is strongly repressed by the global repressor histone like protein (H-NS). Once ToxT is synthesized inside the cell under ToxR regulon [43], H-NS repression is relieved and ToxT directly activates *ctxAB* expression by interacting with RNA polymerase [34]. Previously, both of ToxT and H-NS were found to utilize the heptad repeats for regulating *ctxAB* promoter activity [34, 44]. Interestingly, we found that neither the number of heptad repeats in the CTX promoter region nor the fold change in *ctxB* mRNA expression did correlate with the amount of toxin produced *in vitro*. Any difference in the cellular ToxT and /or H-NS level would have therefore interfered in outcome of the data. Our results with recombinant *V*. *cholerae* and *E*. *coli* strains harboring plasmids; which expressed pre-WT CTB or pre-H20N CTB from *ctxB1* and *ctxB7* respectively, ruled out the possibility that enhanced production of CT by the Haitian variants could be due to any alteration in the virulence cascade regulating *ctxAB* transcription.

By reducing inducible expression of CTB subunits inside the *E coli* cells, we checked whether the fold difference in CTB secretion between the two strains is independent of differential levels in pre-CTB accumulation inside the bacterial cells. It was apparent that the results obtained with 0.2% arabinose induction prevailed even when the inducer dose was gradually dropped up to 0.002% and hence therefore was independent of intracellular accumulation level of the protein. However, as previously reported [35], the expression of cholera toxin genes in *E*. *coli* resulted in the accumulation of cell-adhered toxin subunits. Our observation corroborated the previous findings [36] where existence of an efficient secretory mechanism in *V*. *cholerae* was proposed. This secretion machinery was predicted to be capable of recognizing toxin subunits with an additional secretory step resulting in the release of toxin into the medium. Our results which showed a difference in the CTB accumulation between recombinant *E coli* cells producing WT or Haitian (H20N) pre-CTB, was also seen for the holotoxin secretion. This result supports the proposal given by Hirst and colleagues where it was pointed out that B subunit domains are crucial for assembly and secretion of the holotoxin as they carry the secretory signal for interacting with the protein export machinery. We observed accumulation of mature CTB subunits inside the cells expressing H20N CTB from the *ctxB7* allele, which ultimately leads to elevated toxin subunit secretion in to the media. However, it remained undetermined whether the observed increase in the mature CTB pool was due to efficient pre-H20N CTB processing or simply resulted from increased CTB production from the *ctxB7*. As we could not observe any noteworthy difference between the accumulation levels of *ctxB1* and *ctxB7* mRNAs upon arabinose induction, we predict that the difference in CTB production from the two *ctxB* allelic variants is more likely because of a post transcriptional mechanism underlying CTB synthesis and /or enzymatic processing of pre-CTB monomers. It ought to be mentioned here, however, that since the GM_1_ ELISA technique detects only B subunit oligomers through their interaction with the GM_1_ coated microtiter plates, our data could only measure mature CTB monomers (devoid of the signal sequences) assembled alone or as a part of the CT holotoxin and could not throw any light on the concentration of CTB monomers in the periplasm or in the cytosol. Therefore the amount of pre-CTB monomers translocating across the inner membrane at a given time remains undetected.

As 20%-30% [45–46] of the total cellular proteins in bacterial cells are localized outside the cytosol, it is evident that protein transport across the plasma membrane is a crucial biological process for the survival of cells. Proteins destined for outer cytoplasmic compartments in both eukaryotic and prokaryotic cells typically contain signal peptide, which plays a crucial role in targeting proteins toward the right compartment to fulfil their specific biological functions. Elaborative research works on bacterial signal peptides and their role on transport of proteins out of the cytoplasm had documented importance of the conserved physical features of prokaryotic signal sequences. A typical signal peptide is composed of three well characterized domains: a positively charged amino terminus (N region); the central core (H region) rich in nonpolar, hydrophobic amino acids and the cleavage region (C region) consisting of small, neutral residues [38]. The biophysical properties of these three domains irrespective of the amino acid sequences are well conserved in the diverse signal sequences [47]. Our *in silico* characterization of the CTB signal peptide identified three distinct domains in the CTB signal sequence. One of the hallmark features of natural signal sequences is requirement of a net positive charge in the N-terminus regardless the magnitude of it, which increases transport efficiency [39]. This further supports our result where we observed a dramatic loss in CTB production by the K3E mutant when the net positive charge in the N terminus of CTB signal sequence dropped down to zero. We found formation of an additional β turn in the cleavage region of H20NCTB_SS._ Earlier observations suggested appearance of β turn immediately before or after the cleavage sites [41, 48] and exposed regions in proteins are often found to be associated with turns, or loops [49], which are more flexible and favor accessibility to a given sequence. In view of that we predicted that an additional β -turn formation in the cleavage region of H20NCTB_SS_ could be important for the membrane bound signal peptidase to access the cleavage site.

It ought to be mentioned here that although our study did suggest a definite role of the *ctxB7* allele in increased CT production, we carefully note the limitations and scope of analysis of our work. Since our data are largely derived from ELISA assays, routine variations among standards and samples were observed. Therefore, most reproducible data sets of at least four individual experiments were selected for the statistical analysis. While we are able to quantify the amount of mature CTB subunits assembled inside the cell through semi-quantitative western blot, we could not probe unprocessed precursor fraction in case of *E*. *coli* cells expressing either the pre-WTCTB or pre-H20N CTB from *ctxB1* or *ctxB7*, respectively. This is the reason why we could not measure precursor processing and translocation of pre-CTB monomers across the plasma membrane in either case. Therefore, we have been unable to draw conclusion on whether the mutated signal sequence plays a definite part in pre-H20N precursor processing. There are other possibilities as well. For example, *ctxB7* allele may be more efficiently translated and so on. On the basis of the data the hypothesis that *ctxB7* plays a definitive role in elevated production of CT, seems more plausible. It may also be possible that the *ctxB7* allele may be preferentially or more efficiently translated. Furthermore, cytosolic partner proteins of the Sec-machinery like SecA, may also differentially interact with unprocessed precursor protein (pre-CTB or pre-H20N) through its affinity for the WT or H20N signal sequences, thereby influencing the precursor targeting and translocation. While all of the possibilities remain hypothetical, in summary, our data hints towards higher production of mature CTB subunits at a given time.

Using different modelling approaches, we found that H20NCTB_SS_ formed a β-turn which spans the A19, N20 and G21 amino acid residues around the cleavage region of H20NCTB_SS_. Therefore, considering all of the cumulative data, here we propose a hypothesis which suggests that *ctxB7* may play a pivotal role in elevated CT production. It is possibly due to the structural alteration, which may result in enhanced precursor processing and increased CTB secretion. As the structural information for interacting with the secretion apparatus is contained within the B subunit pentamers, increased secretion of CTB may ultimately result in higher amount of CT production in Haitian variants.

We speculate that adding *β*-turns to CTB signal peptide may increase its processing across the plasma membrane. There is evidence for this, as it was observed that reduction on *β*-turn probabilities lowers the efficiency of translocation by providing flexibility in the choice of other neighbouring cleavage sites [50]. Interestingly, a study by Barkocy-Gallagher [41] showed that signal peptide mutants of maltose binding protein (MBP) with decreased probability of *β*-turn formation in the processing region slowed or eliminated processing.

To understand whether increased β-turn may lead to more efficient cleavage, site-directed mutagenesis can be employed for targeted amino acid substitution with increased probability of β-turn formation near the cleavage region in the signal peptide. This should allow the direct evaluation of the structural or functional importance of for specific signal peptide residues. Furthermore, increased cleavage of pre-CTB may also be quantified by *in vitro* translocation assay with purified components.

There are databases like “MEROPS” and “Brenda” which preserve information resources for peptidases (also termed proteases, proteinases and proteolytic enzymes) and enzymes. Structural-functional data can be retrieved from these databases and computational modelling/docking of a simulated CTB signal peptide can be performed.

Here in this study, we have asked the specific role of the *ctxB7* allele in the hypertoxigenic phenotype of Haitian variant O1 strains which have been associated with various cholera outbreaks in the recent past. The data presented in our paper shows that not only the H20N CTB variant is produced efficiently via post-transcriptional control in clean *E*. *coli* background, but also hyper production is observed in JBK70 as well. It is tempting to speculate that the same mechanism could be operative in *V*. *cholerae* also. Although, our data do not allow us to draw any conclusion on whether this mechanism interacts synergistically with ‘other’ proposed mechanisms of CT production regulation, such as *vieA* and/or *hns*-dependent mechanisms.

The hypothesis that a single amino acid substitution in the signal peptide could result in efficient precursor processing may be of interest to the biochemical community. This may propound a platform for further research on the intricate mechanism underlying membrane translocation and enzymatic processing of precursor CTB. Future studies should be pursued to understand whether enhanced pre-CTB processing may be directly linked with increased CT assembly, resulting in heightened toxin secretion.

## Supporting information

S1 Fig Comparison of CTB secretion by *Vibrio cholerae* JBK70 strain expressing a signal sequence mutant (K3E) of WT CTB from pBAD24 arabinose inducible plasmid. CT production was determined by GM_1_ CT ELISA in triplicate. Mean value of at least three individual experiments (represented by black diamonds) is presented. Standard deviations are indicated with error bars. Unpaired two-tailed student’s t test was used to analyse the statistical significance of the data. (*P value <0.05). The fold difference in CT values (ng/ml/OD_600nm_) is presented by “X”.
(TIF)

S2 Fig Characterization of the CTB signal sequence. Five residue sliding window based representation of the wild type (WT) signal sequence of cholera toxin subunit B from *V*. *cholere* strain O395 (Protein ID:ACP09574.1). Net charge of the peptide at pH 7.8 was calculated using INNOVAGEN Protein Calculator and the grand average of hydropathy (GRAVY) scores for the respective peptides were calculated using Protein GRAVY server. Figure shows distribution of net charge (at pH7.8) and hydrophobicity pattern along its length.
(TIF)

S3 Fig Prediction of the pre-CTB structure using different modelling tools. **(**A) Pre-WT CTB and (B) pre-H20N CTB structures. Both (A) and (B) resulted from RaptorX. From these two structures it was evident that the signal peptide part of the WT structure is quite linear while the H20N structure is making some turns. Although in this modeled structure N20 residues is not associated with any H-bonding with the neighboring residues but a slight conformational change may form turns through stable electrostatic interactions. This assumption is true for the H20N structure resulted from I-TASSER (C) where N20 is capable of making H-bonding with A19 and Y18 (represented by red dotted lines), while the WT structure from I-Tasser doesn’t form any turn but involved in a helix formation (D).
(TIF)
