# Supplementary figures and images for: Deciphering the possible role of ctxB7 allele on higher production of cholera toxin by Haitian variant Vibrio cholerae O1
Source: PLoS Negl Trop Dis. 2020 Apr 1;14(4):e0008128. doi: 10.1371/journal.pntd.0008128 (PMC7112172; doi:10.1371/journal.pntd.0008128)

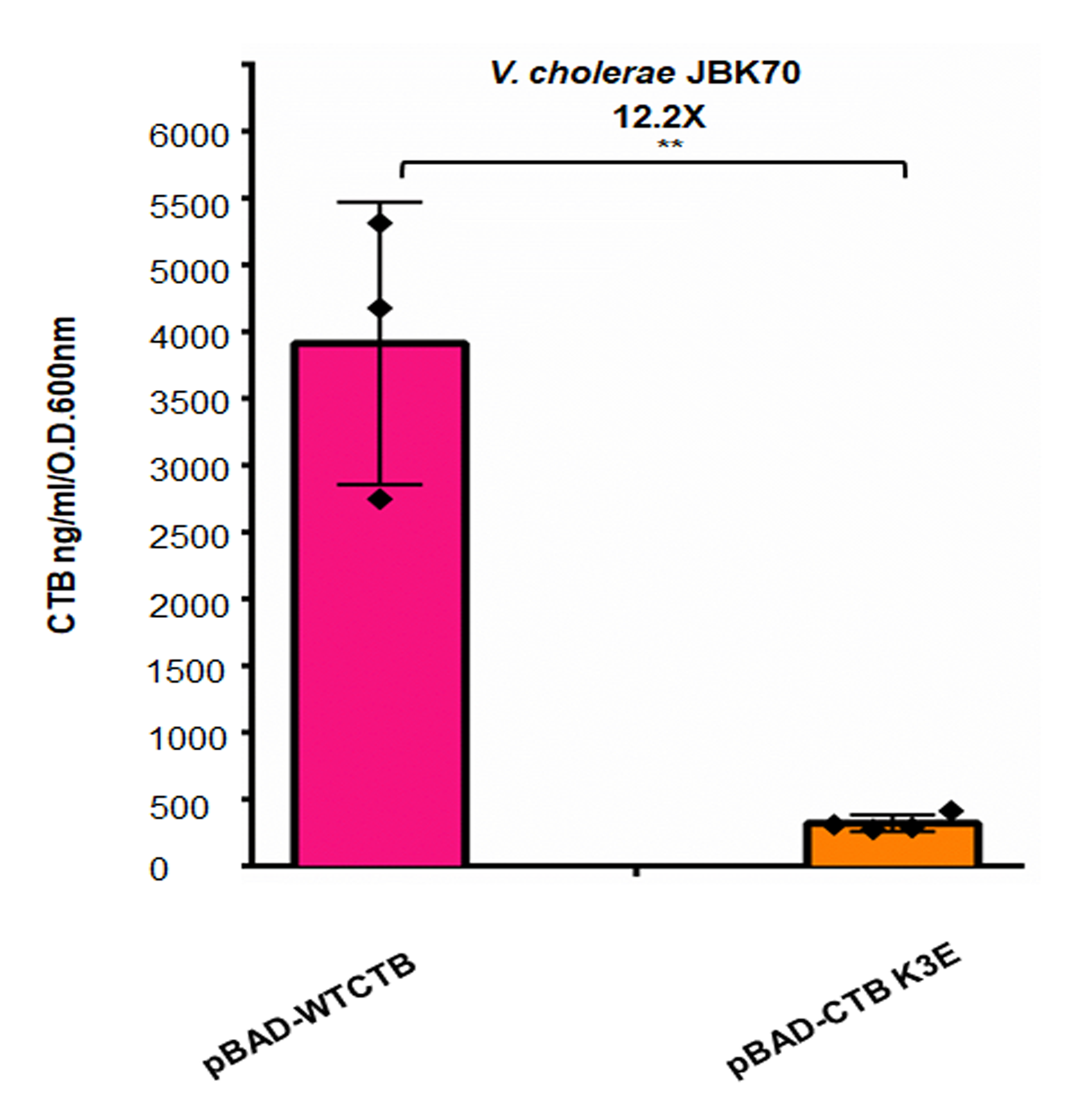

Supplement: S1 Fig — CT production was determined by GM1 CT ELISA in triplicate. Mean value of at least three individual experiments (represented by black diamonds) is presented. Standard deviations are indicated with error bars. Unpaired two-tailed student’s t test was used to analyse the statistical significance of the data. (*P value <0.05). The fold difference in CT values (ng/ml/OD600nm) is presented by “X”. (TIF) [file pntd.0008128.s001.tif]

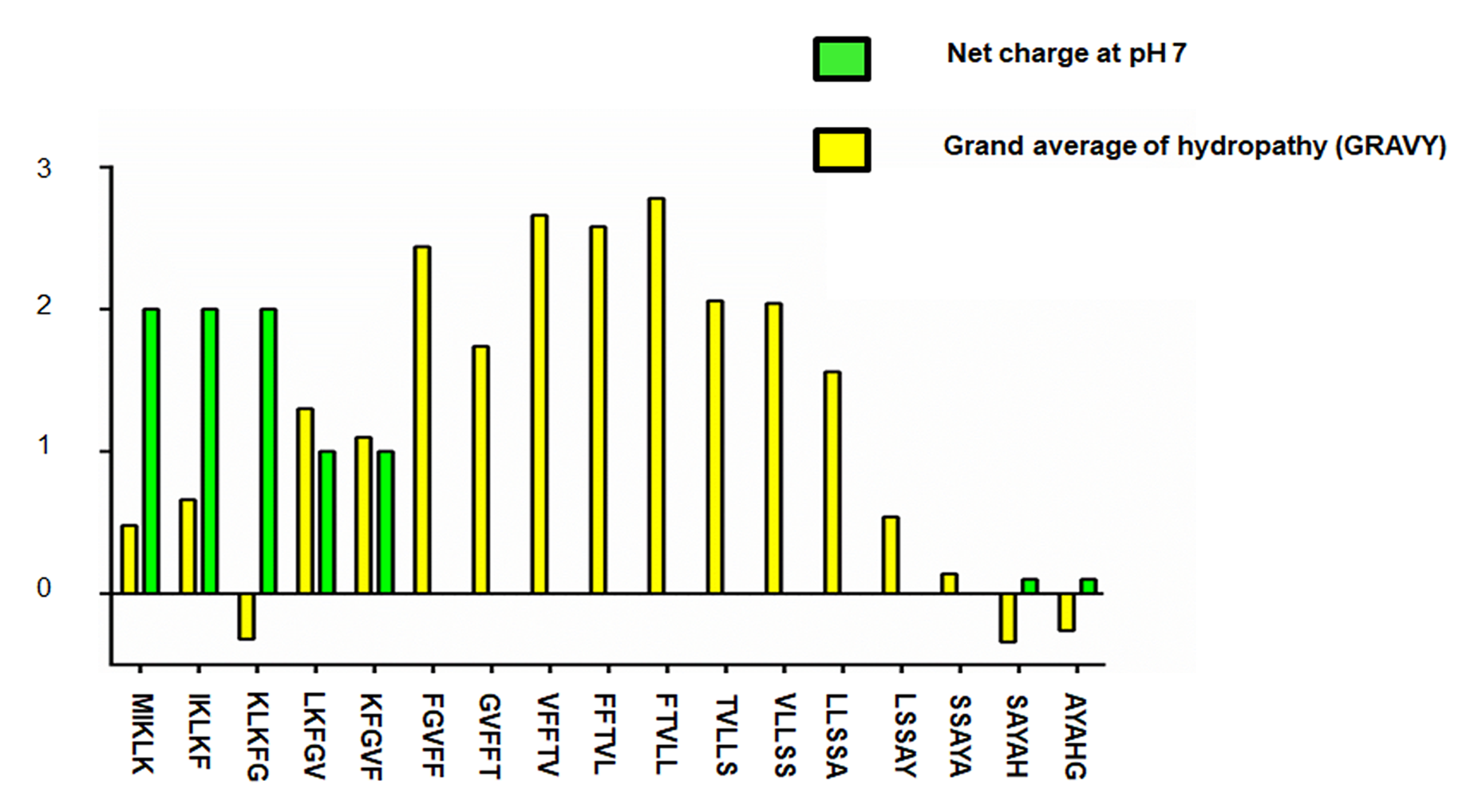

Supplement: S2 Fig — Five residue sliding window based representation of the wild type (WT) signal sequence of cholera toxin subunit B from V. cholere strain O395 (Protein ID:ACP09574.1). Net charge of the peptide at pH 7.8 was calculated using INNOVAGEN Protein Calculator and the grand average of hydropathy (GRAVY) scores for the respective peptides were calculated using Protein GRAVY server. Figure shows distribution of net charge (at pH7.8) and hydrophobicity pattern along its length. (TIF) [file pntd.0008128.s002.tif]

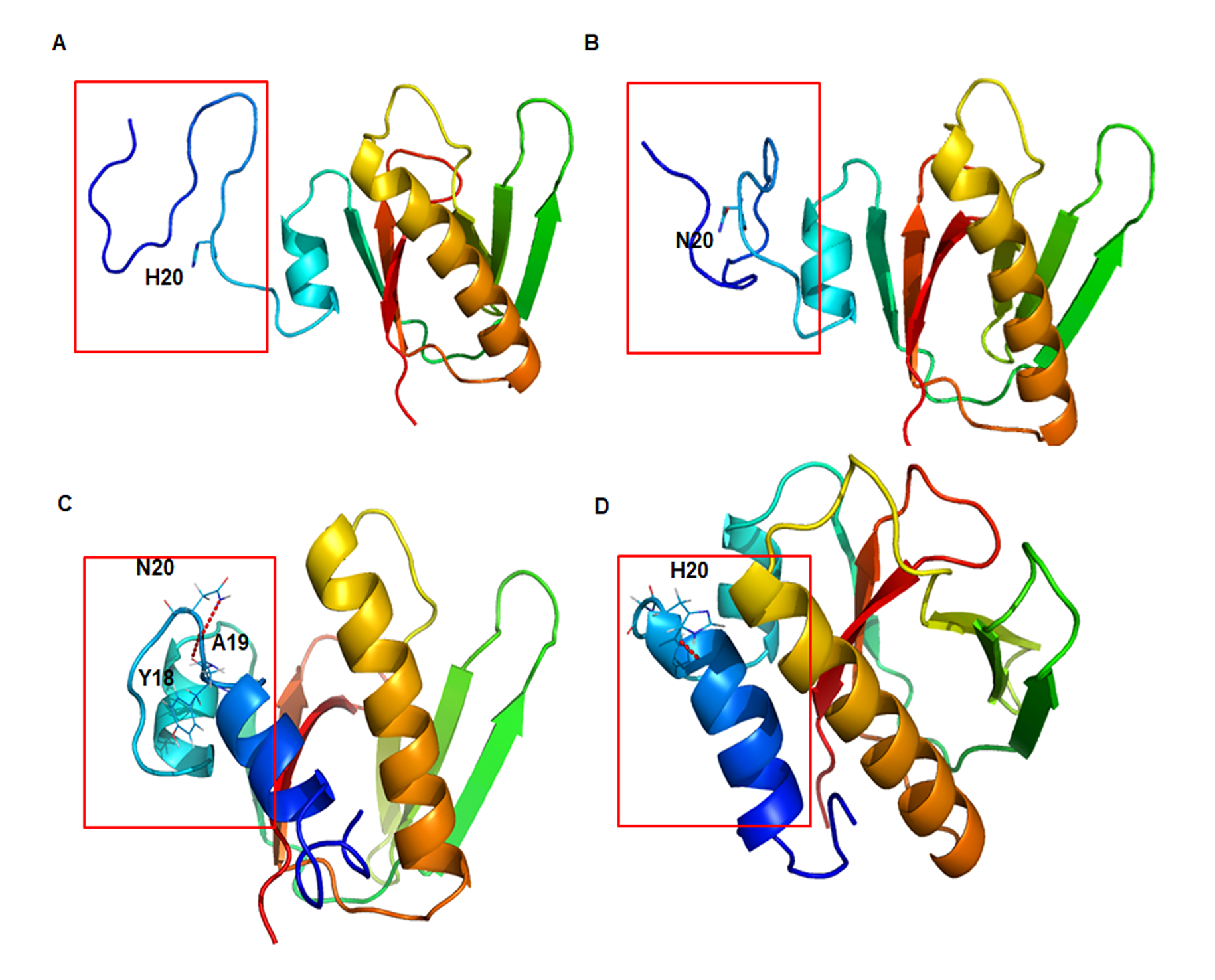

Supplement: S3 Fig — (A) Pre-WT CTB and (B) pre-H20N CTB structures. Both (A) and (B) resulted from RaptorX. From these two structures it was evident that the signal peptide part of the WT structure is quite linear while the H20N structure is making some turns. Although in this modeled structure N20 residues is not associated with any H-bonding with the neighboring residues but a slight conformational change may form turns through stable electrostatic interactions. This assumption is true for the H20N structure resulted from I-TASSER (C) where N20 is capable of making H-bonding with A19 and Y18 (represented by red dotted lines), while the WT structure from I-Tasser doesn’t form any turn but involved in a helix formation (D). (TIF) [file pntd.0008128.s003.tif]
